# Central nitrergic system regulation of neuroendocrine secretion, fluid intake and blood pressure induced by angiotensin-II

**DOI:** 10.1186/1744-9081-6-64

**Published:** 2010-10-25

**Authors:** Wagner L Reis, Wilson A Saad, Luiz A Camargo, Lucila LK Elias, José Antunes-Rodrigues

**Affiliations:** 1 Laboratory of Neuroendocrinology, Department of Physiology, School of Medicine of Ribeirão Preto, University of Sao Paulo USP. Ribeirão Preto, Sao Paulo, Brazil; 2 Department of Physiology, School of Dentistry, Paulista State University of Araraquara. UNESP Araraquara São Paulo, Brazil; 3 Basic Institute of Biosciences, University of Taubaté UNITAU, Taubaté, São Paulo, Brazil; 4 Department of Exact and Natural Science UNIARA Araraquara SP Brazil

## Abstract

**Background:**

Nitric oxide (NO) synthesis has been described in several circumventricular and hypothalamic structures in the central nervous system that are implicated in mediating central angiotensin-II (ANG-II) actions during water deprivation and hypovolemia. Neuroendocrine and cardiovascular responses, drinking behavior, and urinary excretions were examined following central angiotensinergic stimulation in awake freely-moving rats pretreated with intracerebroventricular injections of Nω-nitro-L-arginine methyl ester (L-NAME, 40 μg), an inhibitor of NO synthase, and L-arginine (20 ug), a precursor of NO.

**Results:**

Injections of L-NAME or ANG-II produced an increase in plasma vasopressin (VP), oxytocin (OT) and atrial natriuretic peptide (ANP) levels, an increase in water and sodium intake, mean arterial blood pressure and sodium excretion, and a reduction of urinary volume. L-NAME pretreatment enhanced the ANG-II response, while L-arginine attenuated VP and OT release, thirst, appetite for sodium, antidiuresis, and natriuresis, as well as pressor responses induced by ANG-II.

**Discussion and conclusion:**

Thus, the central nitrergic system participates in the angiotensinergic responses evoked by water deprivation and hypovolemia to refrain neurohypophysial secretion, hydromineral balance, and blood pressure homeostasis.

## Background

Central injections of L-NAME or ANG-II produced an increase in plasma vasopressin (VP), oxytocin (OT) and atrial natriuretic peptide (ANP) levels, an increase in water and sodium intake, mean arterial blood pressure and sodium excretion, and a reduction of urinary volume. L-NAME pretreatment enhanced the ANG-II response, while L-arginine attenuated VP and OT release, thirst, appetite for sodium, antidiuresis, and natriuresis, as well as pressor responses induced by ANG-II. Thus, the central nitrergic system participates in the angiotensinergic responses evoked by water deprivation and hypovolemia by restrain neurohypophysial secretion, hydromineral balance, and blood pressure homeostasis. Nitric oxide (NO) is a lipophilic gas whose synthesis is catalyzed by the enzyme nitric oxide synthase (NOS) from the amino acid L-arginine [1,2]. In the central nervous system, studies have shown that NO plays an important role in neuroendocrine responses, hydromineral balance, and cardiovascular regulation. It may also modulate vasopressin (VP) and oxytocin (OT) release, water and sodium intake/excretion, and arterial blood pressure homeostasis by osmotic and volemic changes.

Water deprivation and hypovolemia stimuli induce a marked activation of the renin-angiotensin system, that increases the circulating level of angiotensin-II (ANG-II) producing physiologic responses including drinking behavior, salt appetite, maintenance of blood pressure, and urinary excretions [3-5]. Intracerebroventricular injection of ANG-II has been found to induce c-fos expression in a restricted number of sites in the forebrain and brainstem, such as neurons in the anterior region of the third ventricle [6,7]. In the central nervous system of rats, the subfornical organ (SFO) is the main site responsible for mediating dipsogenic, natriorexigenic, pressor effects [8], release of VP and OT into the systemic circulation, and renal antidiuretic and natriuretic effects of ANG-II [9-11].

The presence of NOS was described in several brain structures, including the circumventricular system, paraventricular (an important integrator of cardiovascular function regulations), and the supraoptic nuclei, all structures related to central angiotensinergic responses [12,13]. These data suggest the possibility of an interaction between NO and ANG-II in the control of body fluid homeostase NO and ANG-II in the control of body fluid homeostasis. In fact, the expression of NOS gene was increased in the same structures related with ANG-II actions after hypovolemia [14,15] and dehydration [16-18]. Furthermore, the inhibition of endogenous NOS enhances drinking behavior and cardiovascular responses induced by the central administration of ANG II [4,19]. On the other hand, L-arginine, a precursor of NO, as well as NO donors, were able to reduce VP and OT release, water intake, blood pressure, diuretic and natriuretic effects of central angiotensinergic stimulation [4,20-22].

NO induces dipsogenic effect, neurohypophysial secretion, and cardiovascular responses. Under basal normovolemic isosmotic conditions, NO tonically inhibits VP and OT secretion into plasma [23,24]. Thus, in this study we aimed to investigate the role of NO on VP and OT secretion, water and sodium intake/excretion, and blood pressure control following central ANG-II stimulation in rats. This study indicates the variation levels of VP and OT as the NO after angiotensinergic stimulation corresponding to a hydromineral and cardiovascular central regulation.

## Materials and methods

### Animals

Rats were housed in individual cages in a room with controlled temperature (23 ± 2°C) and a 12-12 h light-dark cycle (light on at 6:00 AM) with free access to food pellets and tap water. All the experimental procedures used in these studies were approved by the Ethical Commission of Ethics in Animal Research of the School of Medicine of Ribeirao Preto and by the Medical Ethics Committee of the Paulista State University. Male Wistar rats weighing ~300g were used for hormonal release studies and male Holtzman rats (~280 g) were used for the evaluation of water and saline intake, urinary excretions and blood pressure recording experiments.

### Surgical procedures

For all surgical procedures, Wistar rats were anesthetized with 2.5% 2,2,2-tribromoethanol (Aldrich Chemical Co., USA; 1 ml/100 g body weight), IP) and Holtzman rats were anesthetized with ketamine (80 mg/Kg body weight, IM) and xylazine (7 mg/Kg body weight, IP). Five days before experiments, stainless steel guide cannulas (10.0 mm long, 0.6 mm OD, 0.4 mm ID) were unilaterally implanted into the right lateral cerebral ventricle according to coordinates from the rat brain atlas [25]: 0.6 mm caudal to the bregma, 1.5 mm lateral to the midline and 3.6 mm below the dura mater. The cannula was fixed to the cranium using dental acrylic resin and two jeweler's screws. A 30 gauge metal wire filled the cannula at all times except during the injections. After surgery, the rats received a prophylactic injection of penicillin (50.000 U, IM) and were allowed to recover for 5-6 days, during which time they were handled daily and habituated to the removal of the obturator of the guide cannula to minimize stress during the experimental phase. The correct placement of icv cannula in the lateral ventricle was confirmed at the end of the experiment by injection of Evans Blue (2% in 0.5 μl) into the intracerebroventricular system. Only animals with positive color in the intracerebroventricular system were used in this study. Twenty-four hours before the experiment, rats with an icv guide cannula were re-anesthetized with ketamine and xylazine and a PE-10 polyethylene catheter connected to PE-50 tubing was inserted into the abdominal aorta through the femoral artery for arterial pressure recording. Arterial catheter was tunneled subcutaneously and exposed on the back of the rat to allow access in unrestrained, freely-moving rats.

### Drugs

The drugs were injected into the lateral ventricle using a Hamilton microsyringe connected by a PE-10 polyethylene tube to a needle (30-gauge). L-NAME (Nω-nitro-L-arginine methyl ester) and ANG-II (Asp-Arg-Val-Tyr-Lle-His-Pro-Phe) were purchased from Sigma-Aldrich (St. Louis, MO, USA) and dissolved in isotonic saline (0.15 M NaCl). L-arginine was purchased from Tocris (Ellisville, MO, USA) and dissolved in isotonic saline. The drugs were injected in 0.5 μl volumes over a time period of 30-60 seconds. All doses used in this study were based on previous reports in the literature [4,26].

### Experimental protocols

Twenty four hours before the experiments, all animals were kept in the laboratory and on the day of experiments, they received central administration of isotonic saline (0.15 M NaCl) either alone or containing NOS inhibitor (L-NAME, 40 μg) and NO precursor (L-arginine, 20 μg), followed 10 minutes later, by icv injection of isotonic saline with or without ANG-II (25 ng). The animals were divided into three experimental protocols. The first protocol was designed for the measurement of plasma levels of VP, OT, and ANP. Plasma was collected after the neuropharmacological challenges to determine VO, OT, and ANP concentrations. Five minutes after icv ANG-II injection, animals were decapitated and the trunk blood was collected. The second protocol was designed for the measurement of water and salt intake and sodium and urine excretion. The rats were placed in metabolic cages 24 hours before the experiments and on the next day food was removed. Water and 1.5% NaCl solution were offered during a 60 minute period after ANG-II injection and urinary excretion was collected 120 minutes after ANG-II stimulation. The third protocol was designed to measure arterial blood pressure (MAP). The animals were placed in a test cage and MAP was recorded after 10 minutes resting baseline period and for 30 minutes after icv administration of ANG-II. These protocols are the same for L-NAME and L-arginine alone.

### Determination of plasma hormone levels

Trunk blood was collected into cooled plastic tubes containing heparin for the measurement of VP and OT and into tubes containing EDTA (2 mg/ml of blood) and proteolytic-enzyme inhibitors (20 μl of 1 mM phenylmethylsulfonyl fluoride and 20 μl of 500 μM pepstatin) for the measurement of atrial natriuretic peptide (ANP). Blood samples were centrifuged (1940 g for 20 min at 4°C) and plasma was kept in a freezer at -70°C. For determination of VP and OT, samples were extracted from 1 ml of plasma with acetone and petroleum ether and ANP was extracted from 1 ml of plasma using Sep-Pak C-18 cartridges (Waters Corporation, Milford, MA, USA). Plasma levels of VP, OT, and ANP were measured by specific radioimmunoassays as described previously [27-29]. The assay sensitivity was 7.0 pg/ml for ANP, 0.9 pg/ml for OT, and 0.8 pg/ml for AVP. The inter- and intra-assay variations were 11.7% and 6% for ANP, 12.6% and 7% for OT, and 17.5% and 3.3% for VP, respectively.

### Fluid Intake and Urinary Excretion Measurements

Water and 1.5% NaCl intake were recorded using 0.5 ml graduated burettes (expressed as ml/h). Urinary volume was determined using 100 μl graduated tubes (expressed as ml/2 h). Urinary sodium concentration (expressed as μEq/2 h) was determined by an IL-143 flame spectrophotometer (Instrumental Laboratories, USA).

### Arterial blood pressure recordings

Direct mean arterial blood pressure (MAP) was recorded in unanesthetized and conscious rats (expressed as mmHg). The catheter was connected to a Statham (P23 Db) pressure transducer (Statham-Gould, Valley View, OH) coupled to a multichannel recorder (PowerLab Multirecord, USA), permitting the acquisition of cardiovascular data by computer.

### Statistical analyses

The results are reported as means ± SEM. The data were analyzed by Two-Way ANOVA followed by Newman-Keuls post-hoc test. Statistical analysis was performed using the SigmaStat program. Differences were considered significant at *P *< 0.05.

## Results

### Effects of icv pretreatment with L-NAME and L-ARGININE on VP, OT, and ANP plasma levels induced by central angiotensinergic stimulation

**Figure 1:**
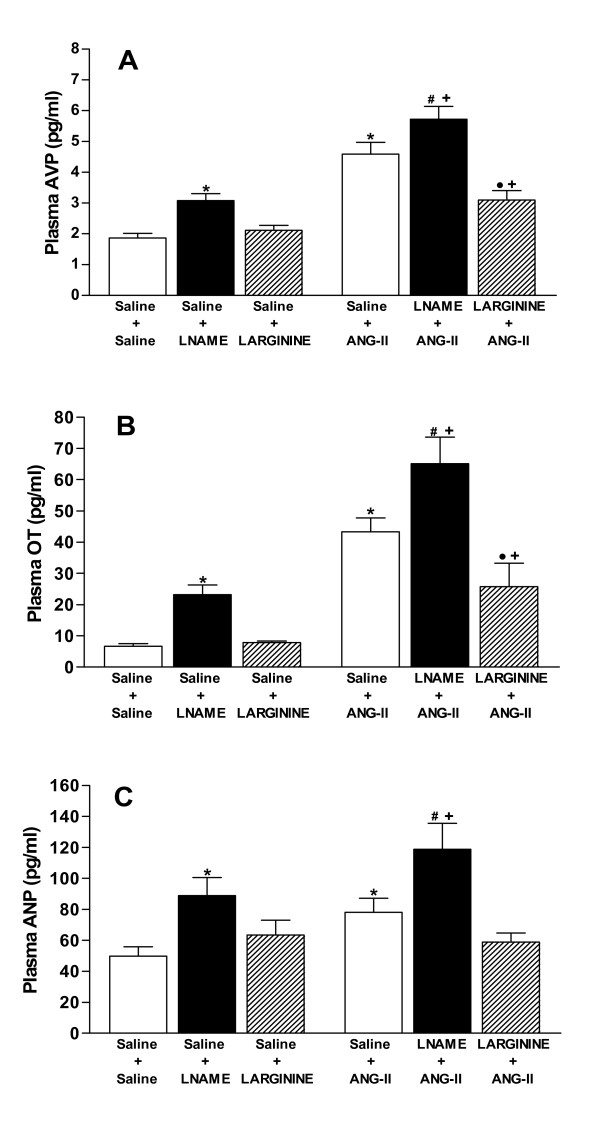
**A, B, C. Effects of pretreatments with isotonic saline (0.9% NaCl), L-NAME (40 μg) or L-ARGININE (20 μg) in rats submitted to central injection of ANG-II (26 ng) or isotonic saline on plasma vasopressin (VP) (A), oxytocin (OT) (B), and atrial natriuretic peptide (ANP) (C) secretion**. Seven to nine animals were used in each group. Differences among groups were determined by Two-Way ANOVA followed by the Newman-Keuls post test. Data are reported as means ± SEM. *P < 0.05 compared to the control group (Saline + Saline). •*P *< 0.05 compared to the Saline+L-ARGININE group. ^*#*^*P *< 0.05 compared to the Saline+L-NAME group. ^*+*^*P *< 0.05 compared to the Saline+ANG-II group.

Figures 1A, B and 1C show the plasma concentration of VP, OT and ANP, respectively. A significant increase (*P *< 0.05) in plasma VP, OT and ANP levels were observed after L-NAME injection compared with the control group (Saline + Saline) of rats. On the other hand, no significant changes were observed in the plasma concentrations of VP, OT and ANP after L-arginine treatment.

The treatment with ANG-II increased (*P *< 0.05) VP, OT and ANP plasma concentrations compared with the control group. In rats stimulated by ANG-II, pretreatment with L-NAME evoked an enhanced (*P *< 0.05) VP, OT and ANP plasma levels above the levels seen when these reagents were injected separately. Conversely, pretreatment with L-arginine reduced (*P *< 0.05) the response of VP and OT concentrations, but it did not modify ANP plasma levels induced by ANG-II.

### Effects of icv pretreatment with L-NAME and L-ARGININE on water and sodium intake induced by central angiotensinergic stimulation

**Figure 2:**
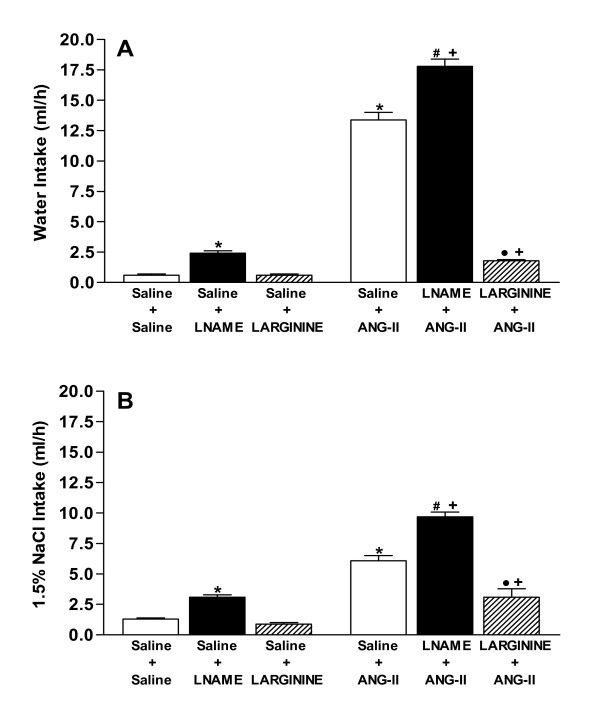
**A, B. Effects of pretreatments with isotonic saline (0.9% NaCl), L-NAME (40 μg) or L-ARGININE (20 μg) in rats submitted to central injection of ANG-II (26 ng) or isotonic saline on water (A) and sodium (B) intake**. Eight to ten animals were used in each group. Differences among groups were determined by Two-Way ANOVA followed by the Newman-Keuls post test. Data are reported as means ± SEM. *P < 0.05 compared to the control group (Saline + Saline). •*P *< 0.05 compared to the Saline+L-ARGININE group. ^*#*^*P *< 0.05 compared to the Saline+L-NAME group. ^*+*^*P *< 0.05 compared to the Saline+ANG-II group.

Figures 2A and 2B show water and sodium chloride intake, respectively. Significant increases (*P *< 0.05) in water and sodium chloride intake were observed after icv injection of L-NAME compared with the control group (Saline + Saline). On the other hand, no significant effects were observed in water and sodium intake after L-arginine injection.

Injection of ANG-II increased (*P *< 0.05) water and sodium intake compared with the control group. Pretreatment with L-NAME in rats who received ANG-II injection potentiated (*P *< 0.05) water and sodium chloride intake values to levels above those observed when the peptide was injected individually. On the contrary, the dipsogenic and natriorexigenic effects of ANG-II were reduced (*P *< 0.05) by pretreatment with L-arginine.

### Effects of icv pretreatment with L-NAME and L-ARGININE on urinary volume and sodium excretion induced by central angiotensinergic stimulation

**Figure 3:**
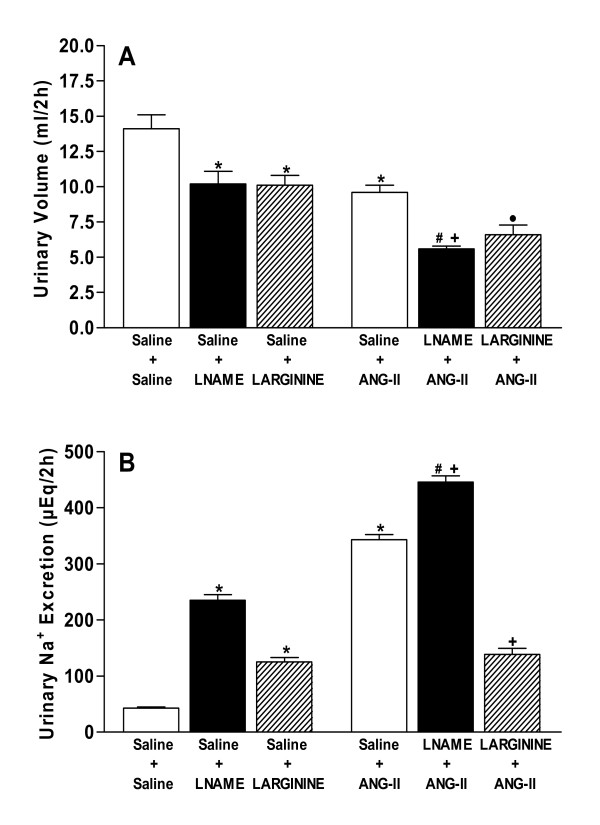
**A, B. Effects of pretreatments with isotonic saline (0.9% NaCl), L-NAME (40 μg) or L-ARGININE (20 μg) in rats submitted to central injection of ANG-II (26 ng) or isotonic saline on urinary volume (A) and sodium (B) excretion**. Eight or nine animals were used in each group. Differences among groups were determined by Two-Way ANOVA followed by the Newman-Keuls post test. Data are reported as means ± SEM. *P < 0.05 compared to the control group (Saline + Saline). •*P *< 0.05 compared to the Saline + L-ARGININE group. ^*#*^*P *< 0.05 compared to the Saline+L-NAME group. ^*+*^*P *< 0.05 compared to the Saline+ANG-II group.

Figures 3A and 3B show urinary volume and sodium excretion, respectively. A significant decrease (*P *< 0.05) in the urine output and an increase in the sodium excretion were observed after injection of L-NAME compared with the control group (Saline+Saline) of rats. On the other hand, a reduction (*P *< 0.05) in the urinary volume and an elevation (*P *< 0.05) in the sodium excretion were observed in rats treated with L-arginine.

Treatment with ANG-II reduced (*P *< 0.05) urinary volume and increased (*P *< 0.05) sodium excretion compared with the control group. In rats stimulated with ANG-II, those pretreated with L-NAME had higher (*P *< 0.05) antidiuresis and natriuresis than that observed when the drugs were injected separately. On the contrary, pretreatment with L-arginine did not modify the urinary volume, although it blocked (*P *< 0.05) the sodium excretion induced by angiotensinergic stimulation.

### Effects of icv pretreatment with L-NAME and L-ARGININE on MAP induced by central angiotensinergic stimulation

**Figure 4:**
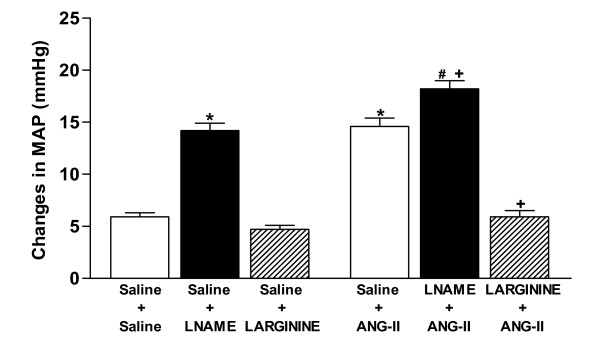
**Effects of pretreatments with isotonic saline (0.9% NaCl), L-NAME (40 μg) or L-ARGININE (20 μg) in rats submitted to central injection of ANG-II (26 ng) or isotonic saline on mean arterial pressure (MAP)**. Eight to ten animals were used in each group. Differences among groups were determined by Two-Way ANOVA followed by the Newman-Keuls post test. Data are reported as means ± SEM. *P < 0.05 compared to the control group (Saline + Saline). *P < 0.05 compared to the control group (Saline+Saline). ^*#*^*P *< 0.05 compared to the Saline+L-NAME group. ^*+*^*P *< 0.05 compared to the Saline+ANG-II group.

Figure 4 shows the MAP response. A significant increase (*P *< 0.05) in mean arterial pressure was observed after icv injection of L-NAME compared with the control group (Saline+Saline). On the other hand, no significant effect was observed after L-arginine injection.

Injection of ANG-II increased (*P *< 0.05) MAP compared with the control group. Rats who were pretreated with L-NAME followed by ANG-II injection had higher (*P *< 0.05) MAP values than those seen when ANG-II and L-name were injected alone. On the contrary, the MAP effect of ANG-II was abolished (*P *< 0.05) by pretreatment with L-arginine. After 30 minutes MAP was restored to control values in all groups.

We founded that there are difference in the change in AVP, OT, ANP plasma levels with L-NAME between the saline and ANG-II groups.

## Discussion

In the present study, we show that the endogenous inhibition of central NO production by a single intracerebroventricular injection of L-NAME promoted an increase of plasma VP, OT, and ANP concentration, MAP water and sodium appetite, and urinary volume and sodium excretion. The effects of the central nitrergic system on hydromineral imbalance in the literature have been controversial, thus, in this study we investigated the effects of NO precursor and NOS blocker after central angiotensinergic stimulation.

Previous studies showed that L-NAME-induced VP, OT, and ANP secretion was correlated with antidiuretic and natriuretic effects [22]. The increase in ANP plasma concentration may be a consequence of OT release [28]. Both hormones act on the kidney closing renal tubules sodium channels and VP is related to water reabsorption in the kidney [30,31]. Thus, our data show that the reduction of central nitrergic tonus induced by NOS blocker may be a result of the predominance of angiotensinergic pathways [19] that facilitate VP and OT release.

We observed that L-NAME induced an increase in MAP; other studies have shown that central treatment with L-NAME increased MAP by reducing NO production in the PVN, an important neural structure that projects to areas in the spinal cord controlling the sympathetic nervous system [19,24]. This effect has been ascribed to a neural component (sympathetic nerve activity) that mediates the short latency and duration of pressor response. Microinjection of NOS blocker directly into the PVN increased the discharges of renal sympathetic nerves, elevating arterial blood pressure [32]. In addition to the neural pathway, there is also a humoral component (vasopressin) responsible for the long duration and latency of the MAP response. The effect of the NOS inhibitor may be mediated by peripheral effects of VP increasing blood pressure [24].

Drinking behavior is one of the most fundamental homeostatic body fluid mechanisms necessary for the maintenance of body fluid balance. The circumventricular organs, including the SFO and organum vasculosum of the laminae terminalis (OVLT) and median preoptic nucleus (MnPO) play an important role in the control of thirst and sodium appetite [33]. In fact, SFO is the main target for ANG-II to stimulate water and salt intake, and it is also involved in the control of blood pressure and neurohypophysial secretion [3,4]. It has been postulated that ANG-II-induced water and sodium intake can be explained, at least in part, by the increase in VP content and neurotransmitter release from axons of neurons onto the effector neurons of drinking responses [34]. Treatment with V1 receptor antagonist was shown to cause a marked decrease in water ingestion, but not sodium appetite, induced by angiotensinergic activation [4].

It is also possible that circumventricular structures are the site of NO action modulating drinking behavior. Several studies have shown that NO is synthesized tonically within the brain during conditions of normal water balance because water and sodium intake increases when endogenous production of NO is inhibited by injection of L-NAME into and around the structures surrounding third ventricle [20,26,35]. In this way, we found that icv injection of L-NAME induces an increase in water and sodium intake.

The results of our experiments show that central angiotensinergic stimulation induced antidiuresis followed by natriuresis associated with an increase of plasma VP, OT, and ANP levels, with a concomitant increase in mean arterial blood pressure and water and sodium intake.

It was previously observed that icv injection of L-NAME in combination with ANG-II enhanced VP and OT secretion [22]. In agreement with these studies, our results show that central pretreatment with L-NAME in rats stimulated with ANG-II can induce a further enhancement not only the VP and OT but also ANP plasma levels with a concomitant increase in the antidiuretic and natriuretic effects. The pressor effect of central ANG-II stimulation was also modulated by L-NAME. There was an initial increase in pressor response within 5 minutes and a prolonged delayed response at 30 minutes [19,24]. In addition, these data show that angiotensinergic stimulation in rats pretreated with L-NAME had potentiated arterial blood pressure and water and sodium intake. Our findings are consistent with other studies, which also showed that central inhibition of NOS enhances ANG-II-induced thirst and sodium appetite [20,26].

In an attempt to elucidate the role of NO on hydromineral, neuroendocrine, and cardiovascular regulation, we also used L-arginine, a precursor of NO, prior to ANG-II stimulation. Our data showed that plasma VP and OT concentration, dipsogenic and natriorexigenic effects, blood pressure, and antidiuretic and natriuretic effects induced by ANG-II were reduced by central pretreatment with L-arginine. It was reported that L-arginine had an antidipsogenic effect when administered centrally to rats deprived of water or stimulated by ANG-II [21]. Other NO donors used in the literature also indicated that the nitrergic system exerts an inhibitory effect on ANG-II-induced VP and OT release and blood pressure [20,22,35]. Therefore, taken together, the present results suggest the inhibitory action of NO system on the effects of ANG-II on neuroendocrine responses, hydromineral balance and blood pressure maintenance.

Besides the central activation of the renin-angiotensin system during hemorrhage and water deprivation, these conditions also evoke an up regulation of NOS gene expression in the circumventricular organs and hypothalamic structures [14-18]. Moreover, systemic or icv infusion of ANG-II promoted an increase of NOS activation and expression in the PVN, SON, SFO, MnPO and OVLT [36-39].

We can hypothesize that there is an interaction between angiotensinergic and nitrergic system in the brain which is required to establish the body fluid homeostasis. ANG-II stimulates NO production, which in turn, counterbalances changes in the VP and OT secretion, blood pressure and water and sodium intake to maintain neurohypophysial hormone store during chronic hypovolemia and dehydration.

The mechanism by which NO can inhibit angiotensin effects is not well understood. Reports in the literature have suggested that NO increases the release of γ-aminobutyric acid (GABA), as shown that bicuculline (GABAergic antagonist), inhibited the neuroendocrine action of NO in the PVN and SON neurons [40-42]. Thus, NO can either reach through indirectly presynaptic neurons in the circumventricular organs or directly postsynaptic PVN and SON neurons, increasing local activation of a GABAergic inhibitory system.

## Conclusion

These results revealed that the endogenous central nitrergic system could influence the ANG-II action in the brain, decreasing neural inputs involved with the neuroendocrine, behavioral, and cardiovascular structures related to the control of osmotic and volemic homeostasis.

## Abbreviations

(NO): Nitric oxide; (ANG-II): Angiotensin-II; (LNAME, 40 μg): Nω-nitro-L-arginine methyl ester L-arginine; (VP): Vasopressin; (OT): Oxytocin; (ANP): Atrial natriuretic peptide.

## Competing interests

The authors declare that they have no competing interests.

## Authors' contributions

WLR and WAS for designed and performed the experiments, analyzed the data and wrote the manuscript. JAR for designed the study, analyzed the data and wrote the manuscript. LLKE for assistance in all steps such as analyses and discussion. LAC for improve the revision version. All authors read and approved the final manuscript.

## References

[B1] Moncada S Palmer RMJ Higgs EA Biosynthesis of nitric oxide from L-arginine: a pathway for the regulation of cell function and communication Biochem Pharmacol 1989 38 1709 1715 10.1016/0006-2952(89)90403-6 2567594

[B2] Bredt DS Snyder SH Nitric oxide: a physiologic messenger molecule Annu Rev Biochem 1994 63 175 195 10.1146/annurev.bi.63.070194.001135 7526779

[B3] Thunhorst RL Johnson AK Renin-angiotensin arterial blood pressure and salt appetite in rats Am J Physiol 1994 266 R458 R465 8141403 10.1152/ajpregu.1994.266.2.R458

[B4] Saad WA Camargo LAA Guarda IFMS Santos TAFB Guarda RS Simões S Saad WA Simões S Antunes-Rodrigues J Interaction between supraoptic nucleus and septal area in the control of water, sodium intake and arterial blood pressure induced by injection of angiotensin II Pharmacol Biochem Behav 2004 77 667 674 10.1016/j.pbb.2004.01.013 15099911

[B5] Schadt JC Ludbrook J Hemodynamic and neurohumoral responses to acute hypovolemia in conscious mammals Am J Physiol 1991 260 H305 18 1671735 10.1152/ajpheart.1991.260.2.H305

[B6] Herbert J Forsling ML Howes SR Stacey PM Shiers HM Regional expression of c-fos antigen in the basal forebrain following intraventricular infusions of angiotensin and its modulation by drinking either water or saline Neuroscience 1992 51 867 882 10.1016/0306-4522(92)90526-8 1488127

[B7] Lauand F Ruginsk SG Rodrigues HL Reis WL de Castro M Elias LL Antunes-Rodrigues J Glucocorticoid modulation of atrial natriuretic peptide, oxytocin, vasopressin and Fos expression in response to osmotic, angiotensinergic and cholinergic stimulation Neuroscience 2007 147 247 257 10.1016/j.neuroscience.2007.04.021 17524563

[B8] Mangiapane ML Simpson JB Subfornical organ: forebrain site of pressor and dipsogenic action of angiotensin II Am J Physiol 1980 239 R382 R389 7435651 10.1152/ajpregu.1980.239.5.R382

[B9] Cunningham ET Jr Sawchenko PE Reflex control of magnocellular vasopressin and oxytocin secretion Trends Neurosci 1991 14 406 411 10.1016/0166-2236(91)90032-P 1720582

[B10] Bastos R Favaretto AL Gutkowska J McCann SM Antunes-Rodrigues J Alpha-adrenergic agonists inhibit the dipsogenic effect of angiotensin II by their stimulation of atrial natriuretic peptide release Brain Res 2001 895 80 88 10.1016/S0006-8993(01)02033-9 11259763

[B11] Saad WA Guarda IF Camargo LAA Santos TAFB Simões S Saad WA Adrenoceptors of the medial septal area modulate water intake and renal excretory function induced by central administration of angiotensin II Braz J Med Biol Res 2002 35 951 959 10.1590/S0100-879X2002000800012 12185387

[B12] Bredt DS Hwang PM Snyder SH Localization of nitric oxide synthase indicating a neural role for nitric oxide Nature 1990 347 768 770 10.1038/347768a0 1700301

[B13] Vincent SR Kimura H Histochemical mapping of nitric oxide synthase in the rat brain Neuroscience 1992 46 755 784 10.1016/0306-4522(92)90184-4 1371855

[B14] Petrov T Harris KH MacTavish D Krukoff TL Jhamandas JH Hypotension induces Fos immunoreactivity in NADPH-diaphorase positive neurons in the paraventricular and supraoptic hypothalamic nuclei of the rat Neuropharmacology 1995 34 509 14 10.1016/0028-3908(95)00002-N 7566485

[B15] Krukoff TL Mactavish D Jhamandas JH Activation by hypotension of neurons in the hypothalamic paraventricular nucleus that project to the brainstem J Comp Neurol 1997 385 285 96 10.1002/(SICI)1096-9861(19970825)385:2<285::AID-CNE7>3.0.CO;2-Y 9268128

[B16] Ueta Y Levy A Chowdrey HS Lightman SL Water deprivation in the rat induces nitric oxide synthase (NOS) gene expression in the hypothalamic paraventricular and supraoptic nuclei Neurosci Res 1995 23 317 9 10.1016/0168-0102(95)00956-6 8545081

[B17] O'Shea RD Gundlach AL Food or water deprivation modulate nitric oxide synthase (NOS) activity and gene expression in rat hypothalamic neurones: correlation with neurosecretory activity? J Neuroendocrinol 1996 8 417 25 10.1046/j.1365-2826.1996.04682.x 8809671

[B18] Srisawat R Bishop VR Bull PM Douglas AJ Russell JA Ludwig M Leng G Regulation of neuronal nitric oxide synthase mRNA expression in the rat magnocellular neurosecretory system Neurosci Lett 2004 369 191 6 10.1016/j.neulet.2004.07.045 15464263

[B19] Bains JS Ferguson AV Angiotensin II neurotransmitter actions in paraventricular nucleus are potentiated by nitric oxide synthase inhibitor Reg Pept 1994 50 53 59 10.1016/0167-0115(94)90191-0 7512740

[B20] Saad WA Guarda IF Camargo LA Garcia G Gutierrez LI Saad WA Simões S Guarda RS Lateral hypothalamus lesions influences water and salt intake, and sodium and urine excretion, and arterial blood pressure induced by L-NAME and FK 409 injections into median preoptic nucleus in conscious rats Life Sci 2004 75 685 697 10.1016/j.lfs.2004.01.018 15172178

[B21] Calapai G Caputi AP Nitric oxide and drinking behaviour Regul Pept 1996 66 117 121 10.1016/0167-0115(96)00050-X 8899905

[B22] Reis WL Giusti-Paiva A Ventura RR Margatho LO Gomes DA Elias LL Antunes-Rodrigues J Central nitric oxide blocks vasopressin, oxytocin and atrial natriuretic peptide release and antidiuretic and natriuretic responses induced by central angiotensin II in conscious rats Exp Physiol 2007 92 903 911 10.1113/expphysiol.2007.037911 17513344

[B23] Liu H Terrell ML Bui V Summy-Long JY Kadekaro M Nitric oxide control of drinking, vasopressin and oxytocin release and blood pressure in dehydrated rats Physiol Behav 1998 63 763 769 10.1016/S0031-9384(97)00528-3 9617997

[B24] Kadekaro M Summy-Long JY Centrally produced nitric oxide and the regulation of body fluid and blood pressure homeostases Clin Exp Pharmacol Physiol 2000 27 450 459 10.1046/j.1440-1681.2000.03264.x 10831252

[B25] Paxinos G Watson C The Rat Brain in Stereotaxic Coordinates 1997 New York, USA: Academic Press

[B26] Saad WA Guarda IF Camargo LA dos Santos TA Saad WA Functional relationship between subfornical organ cholinergic stimulation and nitrergic activation influencing cardiovascular and body fluid homeostasis Regul Pept 2007 143 28 33 10.1016/j.regpep.2007.01.013 17395280

[B27] Elias LL Antunes-Rodrigues J Elias PC Moreira AC Effect of plasma osmolality on activity-adrenal responses to corticotropin-releasing hormone and atrial natriuretic peptide changes in central diabetes insipidus J Clin Endocrinol Metab 1997 82 1243 1247 10.1210/jc.82.4.1243 9100602

[B28] Haanwinckel MA Elias LK Favaretto AL Gutkowska J McCann SM Antunes-Rodrigues J Oxytocin mediates atrial natriuretic peptide release and natriuresis after volume expansion in the rat Proc Natl Acad Sci USA 1995 92 7902 7906 10.1073/pnas.92.17.7902 7644511 PMC41254

[B29] Gutkowska J Thibault G Januszewicz P Cantin M Genest J Direct radioimmunoassay of atrial natriuretic factor Biochem Biophys Res Commun 1984 122 593 601 10.1016/S0006-291X(84)80074-1 6235810

[B30] Verbalis JG Mangione MP Stricker EM Oxytocin produces natriuresis in rats at physiological plasma concentrations Endocrinology 1991 128 1317 1322 10.1210/endo-128-3-1317 1847854

[B31] Nielsen S Chou CL Marples D Christensen EI Kishore BK Knepper MA Vasopressin increases water permeability of kidney collecting duct by inducing translocation of aquaporin-CD water channels to plasma membrane Proc Natl Acad Sci USA 1995 92 1013 1017 10.1073/pnas.92.4.1013 7532304 PMC42627

[B32] Zhang K Mayhan WG Patel KP Nitric oxide within the paraventricular nucleus mediates changes in renal sympathetic nerve activity Am J Physiol 1997 273 R864 R872 9321861 10.1152/ajpregu.1997.273.3.R864

[B33] Antunes-Rodrigues J Castro M Elias LLK Valença MM McCann SM Neuroendocrine control of body fluid metabolism Physiol Rev 2004 84 169 208 10.1152/physrev.00017.2003 14715914

[B34] De-Angelis PR Antunes VR Camargo GM Saad WA Renzi A Camargo LA Central interaction between atrial natriuretic peptide and angiotensin II in the control of sodium intake and excretion in female rats Braz J Med Biol Res 1996 29 1671 1674 9222431

[B35] Saad WA Guarda IF Camargo LA Santos TA Nitric oxide and L-type calcium channel influences the changes in arterial blood pressure and heart rate induced by central angiotesin II Behav Brain Funct 2008 4 22 10.1186/1744-9081-4-22 18501011 PMC2409355

[B36] Ye S Nosrati S Campese VM Nitric oxide (NO) modulates the neurogenic control of blood pressure in rats with chronic renal failure (CRF) J Clin Invest 1997 99 540 8 10.1172/JCI119191 9022090 PMC507830

[B37] Zhu B Herbert J Angiotensin II interacts with nitric oxide-cyclic GMP pathway in the central control of drinking behaviour: mapping with c-fos and NADPH-diaphorase Neuroscience 1997 79 543 53 10.1016/S0306-4522(96)00686-0 9200737

[B38] Dawson CA Jhamandas JH Krukoff TL Activation by systemic angiotensin II of neurochemically identified neurons in rat hypothalamic paraventricular nucleus J Neuroendocrinol 1998 10 453 9 10.1046/j.1365-2826.1998.00225.x 9688348

[B39] Zhang L Tong M Xiao M Li L Ding J Nitric oxide mediates feedback inhibition in angiotensin II-induced upregulation of vasopressin mRNA Peptides 2009 30 913 7 10.1016/j.peptides.2009.01.024 19428769

[B40] Bains JS Ferguson AV Nitric oxide regulates NMDA-driven GABAergic inputs to type I neurones of the rat paraventricular nucleus J Physiol 1997 499 733 46 9130169 10.1113/jphysiol.1997.sp021965 PMC1159291

[B41] Zhang K Patel KP Effect of nitric oxide within the paraventricular nucleus on renal sympathetic nerve discharge: role of GABA Am J Physiol 1998 275 R728 34 9728069 10.1152/ajpregu.1998.275.3.R728

[B42] Stern JE Ludwig M NO inhibits supraoptic oxytocin and vasopressin neurons via activation of GABAergic synaptic inputs Am J Physiol 2001 280 R1815 R1822 10.1152/ajpregu.2001.280.6.R1815 11353687

